# Author Correction: Extensive genomic diversity among *Mycobacterium marinum* strains revealed by whole genome sequencing

**DOI:** 10.1038/s41598-020-61218-5

**Published:** 2020-03-18

**Authors:** Sarbashis Das, B. M. Fredrik Pettersson, Phani Rama Krishna Behra, Amrita Mallick, Martin Cheramie, Malavika Ramesh, Lisa Shirreff, Tanner DuCote, Santanu Dasgupta, Don G. Ennis, Leif. A. Kirsebom

**Affiliations:** 1 Department of Cell and Molecular Biology, Box 596, Biomedical Centre, SE-751 24 Uppsala, Sweden; 2 Department of Biology, University of Louisiana, Lafayette, Louisiana USA

Correction to: *Scientific Reports* 10.1038/s41598-018-30152-y, published online 13 August 2018

The Article contains an error in Table 1 where the ‘Host’ column for the strain ‘DE4576/Huestis’ is incorrectly listed.

“Zebrafish (*Danio rerio*), outbreak of the ZIRC National zebrafish facility”

should read:

“Zebrafish (*Danio rerio*), outbreak of a laboratory facility, University of Oregon”

Additionally, in the ‘Isolated from’ column for the strain ‘DE4576/Huestis’, the city ‘Eugene’ is missing.

“Oregon, USA”

should read:

“Eugene, Oregon, USA”

