# Supplementary material for: Extensive genomic diversity among Mycobacterium marinum strains revealed by whole genome sequencing
Source: Sci Rep. 2018 Aug 13;8:12040. doi: 10.1038/s41598-018-30152-y (PMC6089878; doi:10.1038/s41598-018-30152-y)
Supplement: Supplementary file 1 — Supplementary information [file 41598_2018_30152_MOESM1_ESM.pdf]

Supplementary information

Extensive genomic diversity among *Mycobacterium marinum* strains revealed by whole genome sequencing

Sarbashis Das[1], B. M. Fredrik Pettersson[1], Phani Rama Krishna Behra[1], Amrita Mallick[2], Martin Cheramie[2], Malavika Ramesh[1], Lisa Shirreff[2], Tanner DuCote[2], Santanu Dasgupta[1], Don G. Ennis[2] and Leif. A. Kirsebom*[,1]

[1]Department of Cell and Molecular Biology
Box 596, Biomedical Centre
SE-751 24 Uppsala, Sweden

[2]Department of Biology,
University of Louisiana,
Lafayette, Louisiana, USA

*corresponding author

Leif.kirsebom@icm.uu.se

Supplementary Figure-1:

a

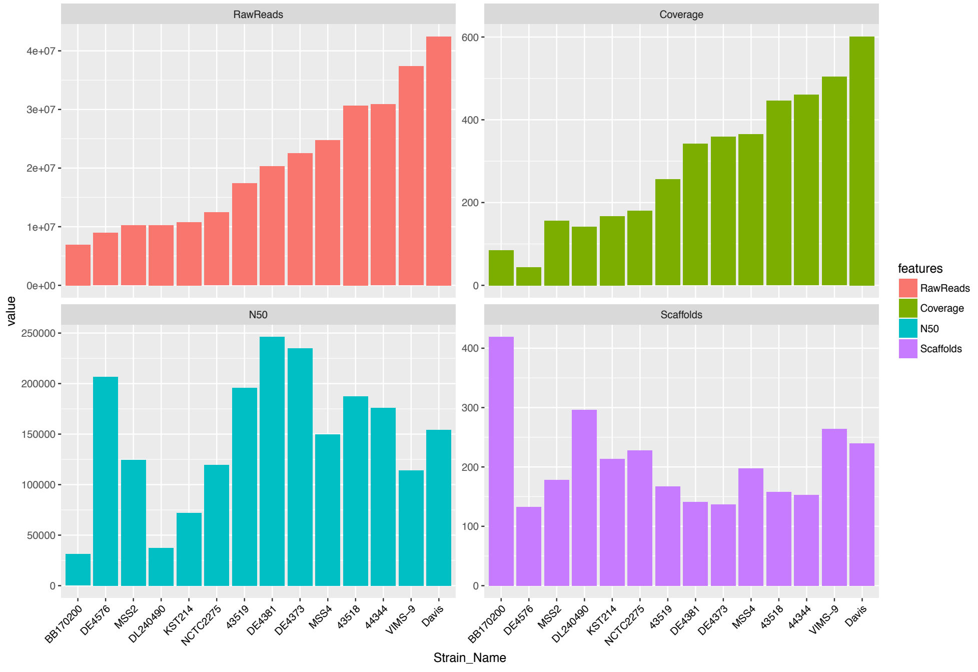

b

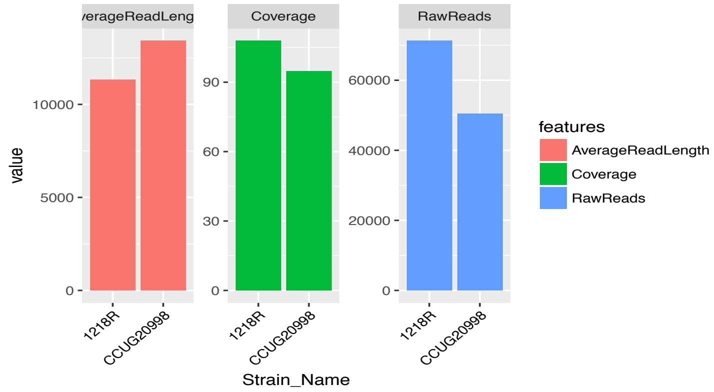

Supplementary Figure S1 Genome sequencing reads and assembly statistics

(a) Bar plots showing number of raw reads, coverage, N50 and number of scaffolds in the 14 *Mma* genomes sequenced using Illumina sequencing platform.

(b) Plots showing average read lengths, coverage, and raw reads for the 1218R and CCUG20998 genomes sequenced using Pacific Biosciences technology.

Figure-S2:

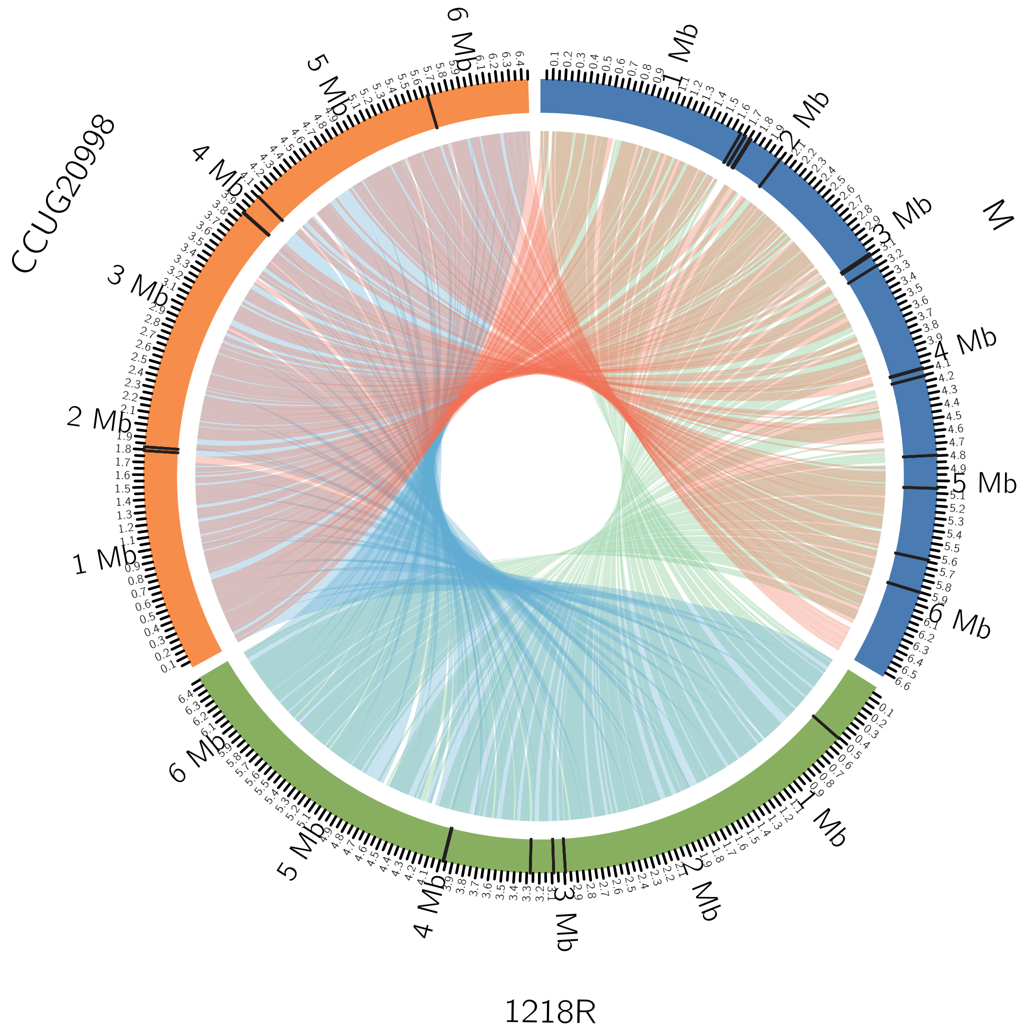

Supplementary Figure S2 Genome wide distribution of IS elements in complete genomes

Circos plot of the genome alignment of three complete genomes: M, 1218R and CCUG20998 represented by blue, green and orange arcs, respectively. Lines connecting the arcs show homologous regions in the genome while white gaps between the lines indicate unique genomic regions. Predicted IS elements are marked with black bars in the corresponding genomes. Scales outside the arcs shows the lengths of the genomes.

Figure-S3:

a.

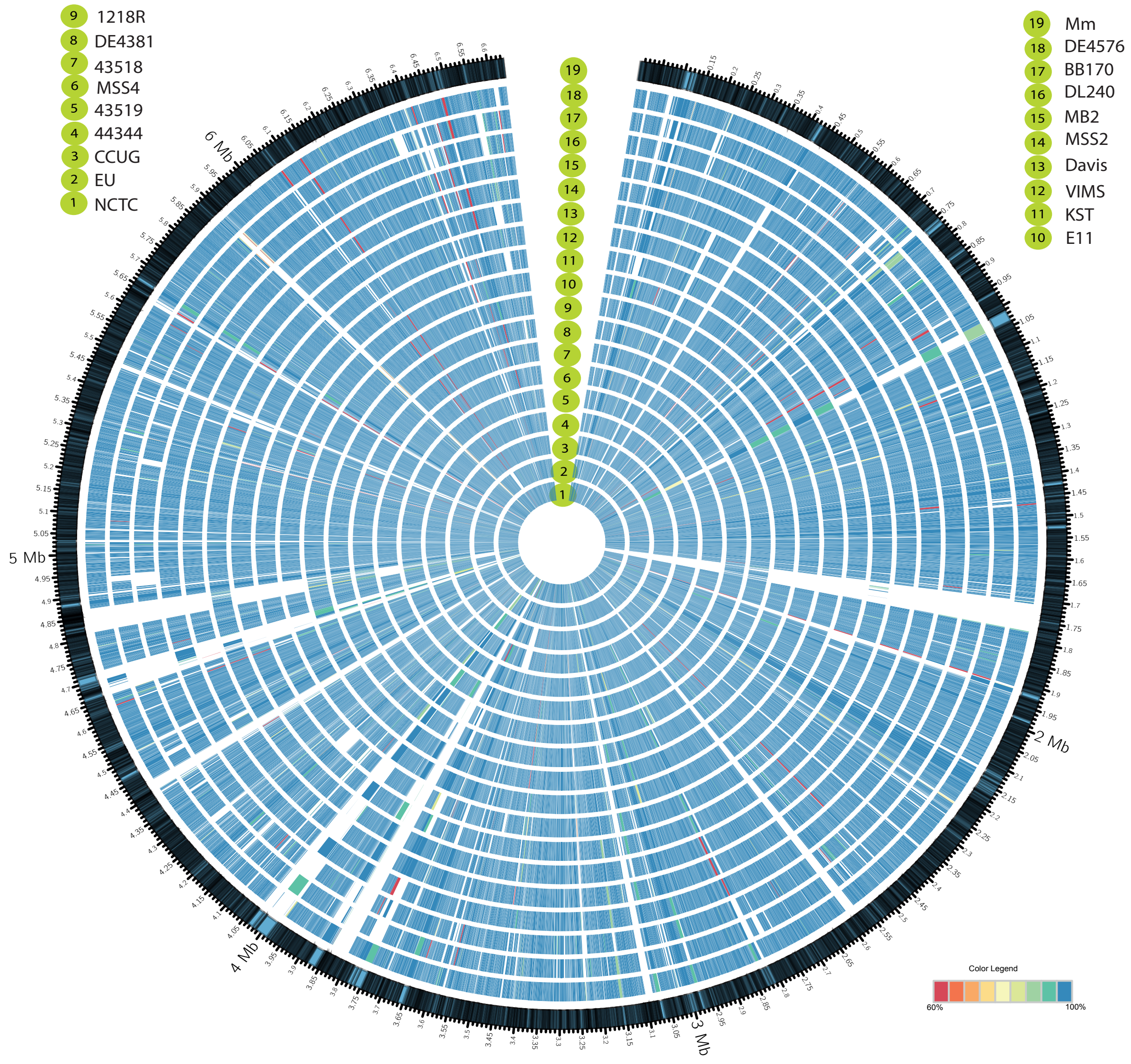

b.

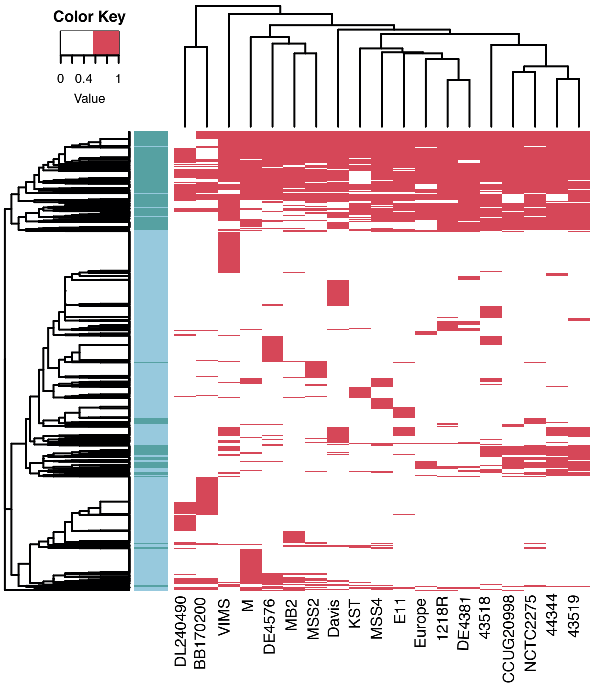

c.

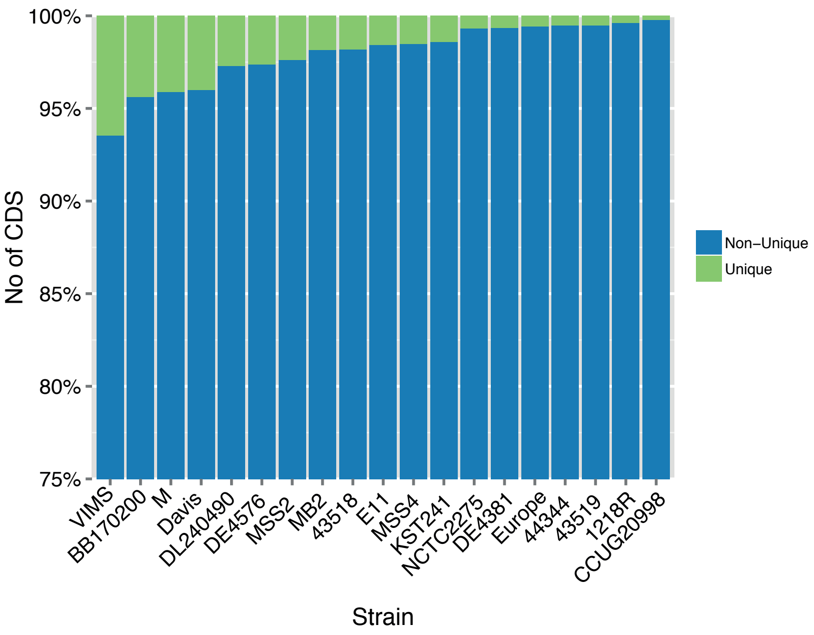

Supplementary Figure S3 *Mma* M strain orthologous and non-core genes

(a) Circos plot showing the presence of protein coding genes in 18 *Mma* strains compared to the M strain. The outer track represents the genome for the M strain with a size scale. Radial black lines with blue fill mark genes in the M strain. Each circular track represents one genome and the number corresponds to the strain name in the legend. Coloured radial blocks represent orthologous genes in the corresponding genome and colour intensity indicates percentage identity at the protein levels. The white blocks indicate that no orthologs were identified. (b) Heat map showing presence (red) and absence (white) of orthologous genes in the different *Mma* strains. Clustering of the values was done using hierarchical clustering. (c) Stacked bar plot showing the percentage of unique and non-unique genes in all *Mma* strains.

Figure-S4:

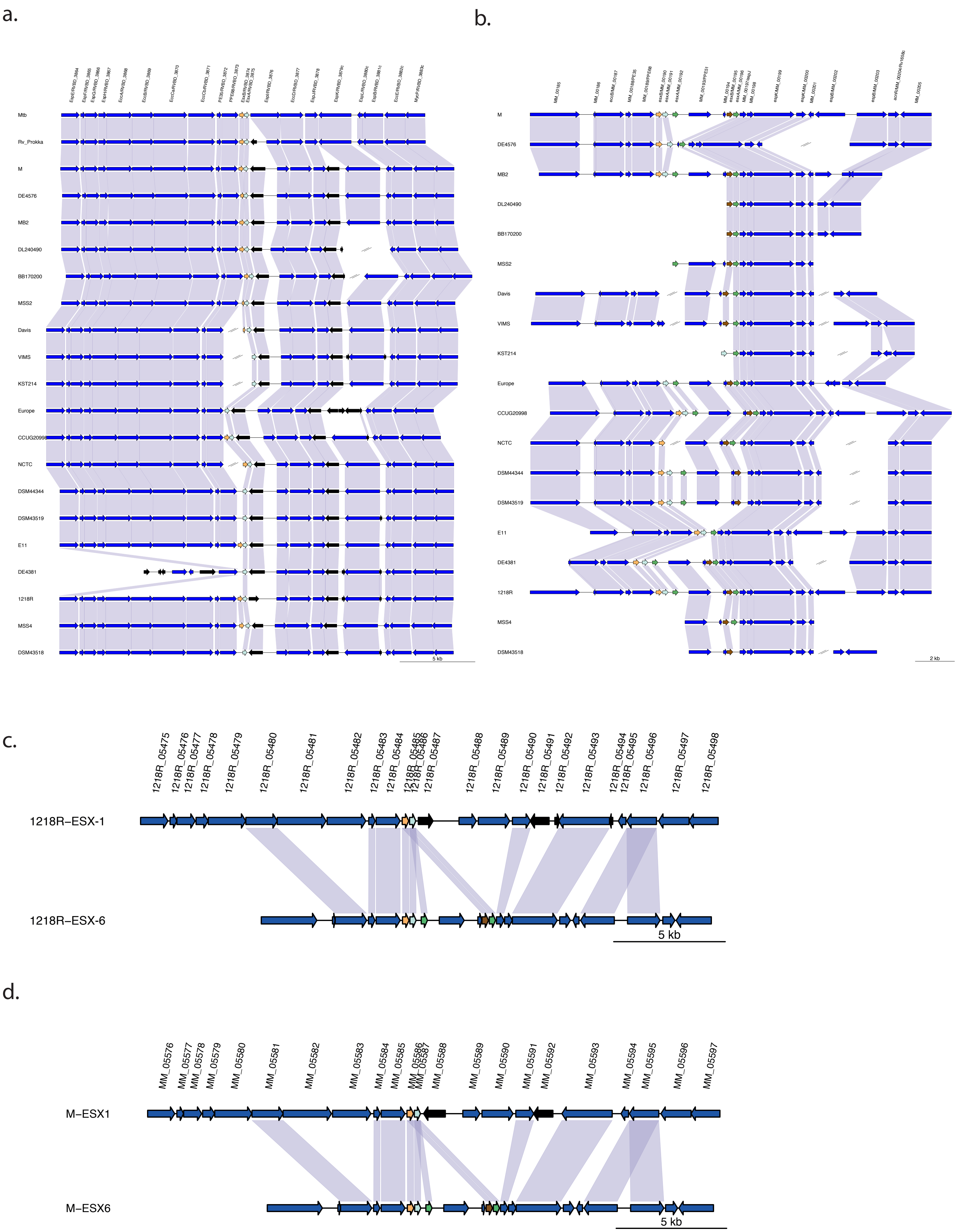
a.
Mtb
Rv_Prokka
M
DE4576
MB2
DL240490
BB170200
MSS2
Davis
VIMS
KST214
Europe
CCUG20998
NCTC
DSM44344
DSM43519
E11
DE4381
1218R
MSS4
DSM43518
5 kb
b.
M
DE4576
MB2
DL240490
BB170200
MSS2
Davis
VIMS
KST214
Europe
CCUG20998
NCTC
DSM44344
DSM43519
E11
DE4381
1218R
MSS4
DSM43518
2 kb
c.
1218R_05475
1218R_05476
1218R_05477
1218R_05478
1218R_05479
1218R_05480
1218R_05481
1218R_05482
1218R_05483
1218R_05484
1218R_05485
1218R_05486
1218R_05487
1218R_05488
1218R_05489
1218R_05490
1218R_05491
1218R_05492
1218R_05493
1218R_05494
1218R_05495
1218R_05496
1218R_05497
1218R_05498
1218R–ESX-1
1218R–ESX-6
5 kb
d.
MM_05576
MM_05577
MM_05578
MM_05579
MM_05580
MM_05581
MM_05582
MM_05583
MM_05584
MM_05585
MM_05586
MM_05587
MM_05588
MM_05589
MM_05590
MM_05591
MM_05592
MM_05593
MM_05594
MM_05595
MM_05596
MM_05597
M–ESX1
M–ESX6
5 kb

Supplementary Figure S4 Gene synteny plot of ESX-1 regular and partially duplicated gene clusters in different *Mma* strains

Gene synteny plot showing (a) complete ESX-1 and (b) partially duplicated ESX-6 gene clusters in all the *Mma* strains. Arrows represent genes and their direction indicates strand information while vertical connections indicate orthologous genes. Genes are drawn to scale. Color code: blue (ESX-1 related genes) and black (hypothetical protein) arrow mean upstream/downstream of *esxB* and *esxA* loci. The regular *esxB* and *esxA* genes are coloured as yellow and light green respectively and the connecting light blue shaded vertical line between the arrows indicate homologous genes. The *Mtb*H37Rv genome was re-annotated (marked as Rv_Prokka) using the same approach as used for annotation of the other genomes. For comparison, we included the annotation of *Mtb*H37Rv (marked Mtb in the figure) used by Stinear et al. (2008)[6]. Comparing the two annotations revealed that the original EspI/RVBD_3876 gene is annotated as two genes as predicted to be the case in the *Mma* genomes. In (b) the regular *esxB* and *esxA* orthologs are coloured in yellow and light green while the respective paralogs are coloured in brown and green. The genes marked in shaded light blue indicate homologous genes. (c) Heat map showing presence and absence of *esxA* and *esxB* orthologous and paralogous in 1218R. (d) Similar to (c) but for the M strain.

Figure-S5:

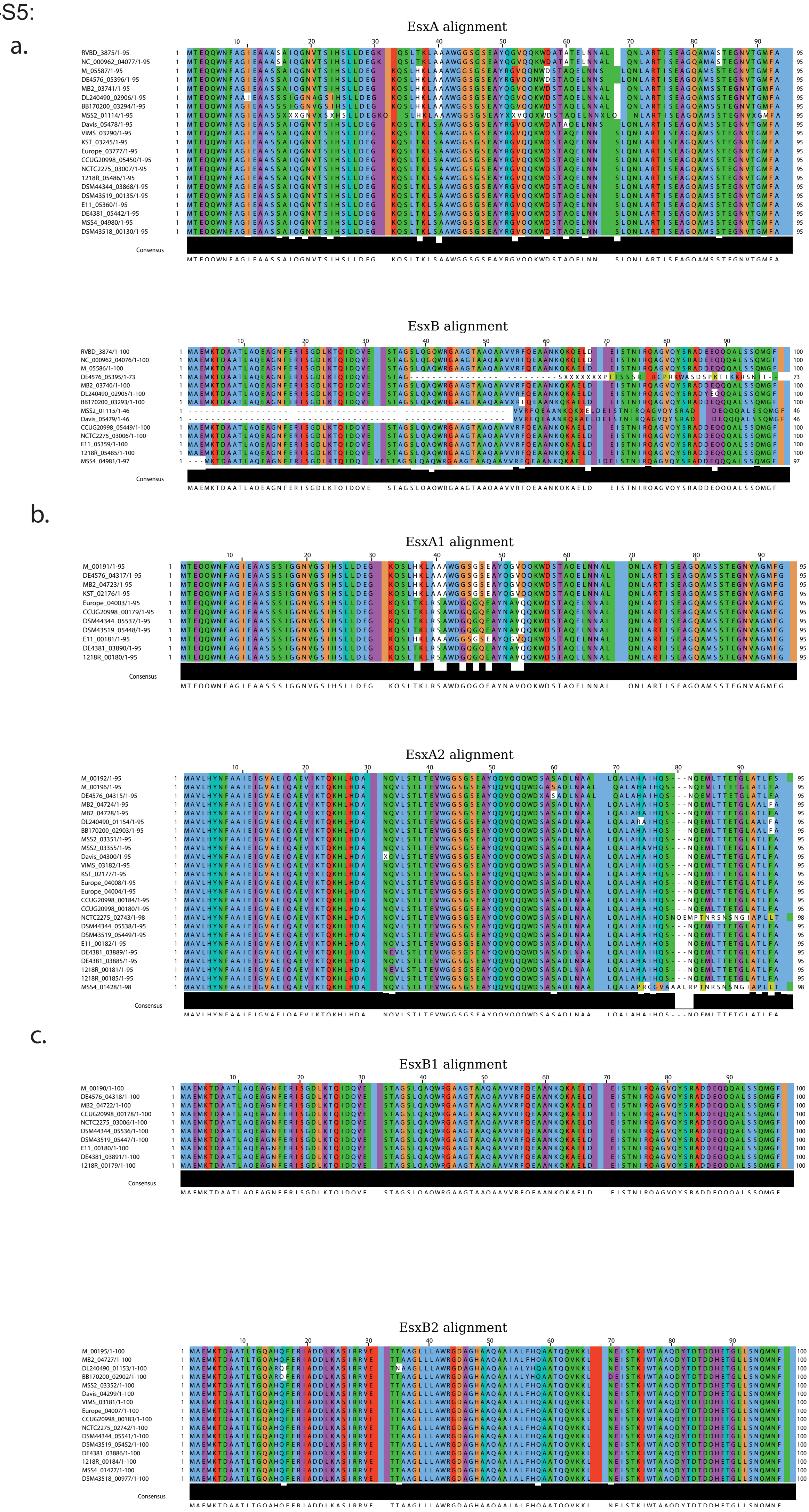
a.
EsxA alignment
EsxB alignment
b.
EsxA1 alignment
EsxA2 alignment
c.
EsxB1 alignment
EsxB2 alignment

Supplementary Figure S5 Multiple sequence alignments: EsxA and EsxB and their orthologs in the *Mma* strains

(a) Top, alignment showing EsxA and its orthologous genes while the bottom alignment represents EsxB and its orthologs in the *Mma* strains.

(b) Similar to a, top alignment for EsxA1 and bottom showing EsxA2.

(c) Similar to a, b and the top alignment show EsxB1 and bottom EsxB2.

Figure-S6:

a.

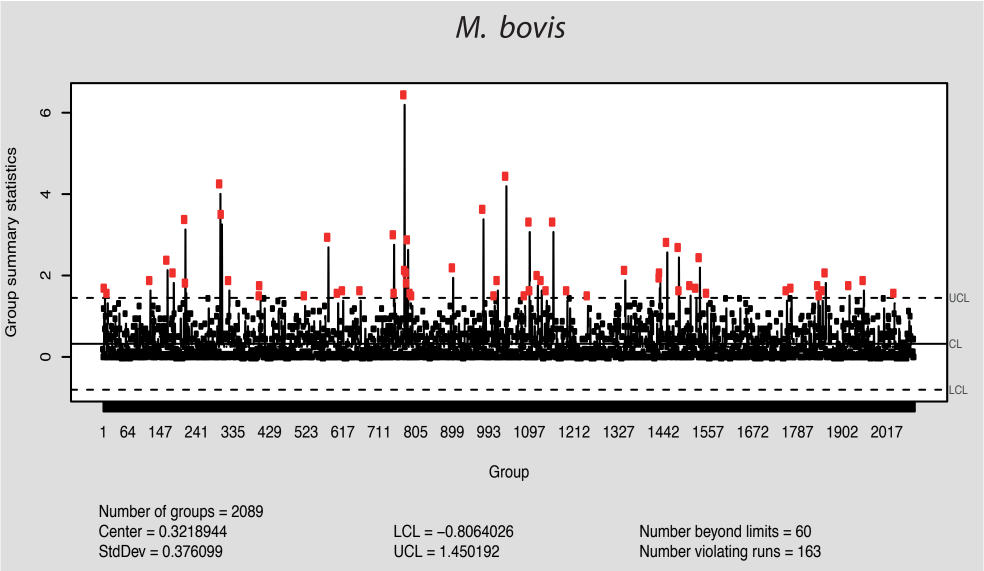

b.

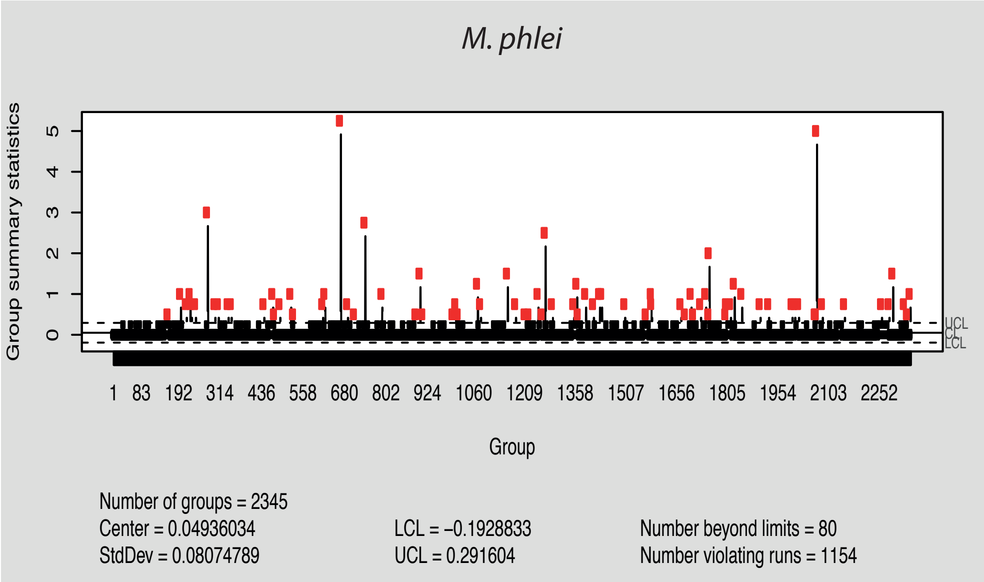

c.

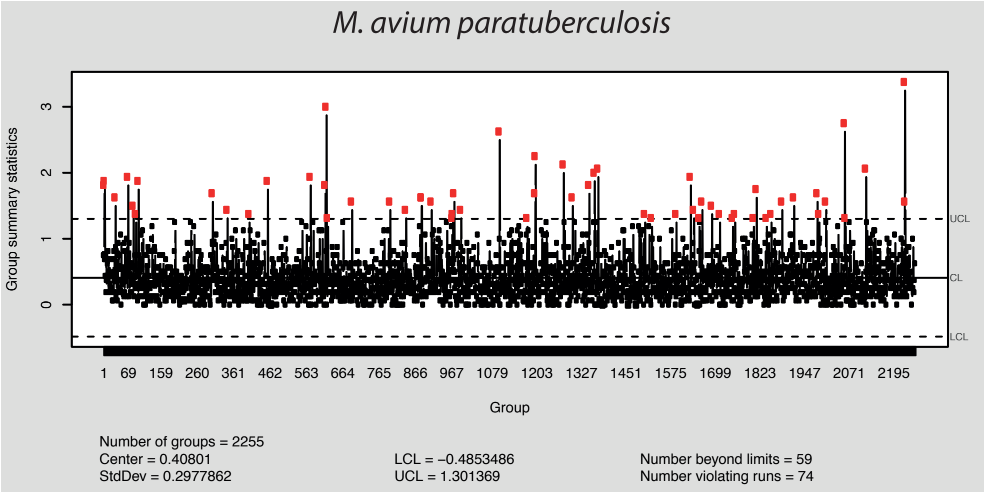

Supplementary Figure S6 Analysis of mutational hotspots in other mycobacteria

Shewhart control chart showing average SNVs frequencies for different mycobacteria as indicated. Red and black dots mark out of control (hotspots) and in-control SNV frequencies, respectively.

(a) 26 strains of *Mbo* (covering both *M. bovis* and *M. bovis* BCG genomes).

(b) Five *Mph* strains[34].

(c) 23 MAP strains

The different genomes were extracted from the NCBI database.

Figure-S7:

a

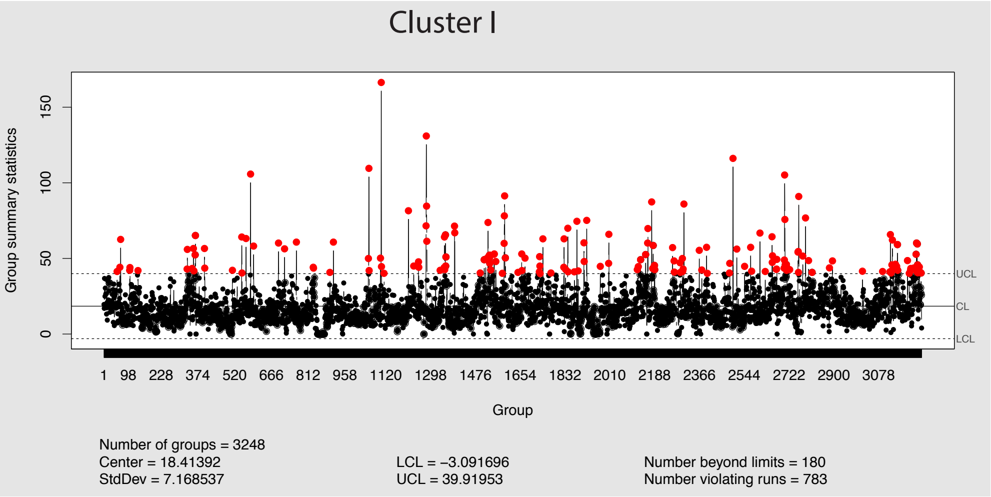
Cluster I
Group summary statistics
0
50
100
150
UCL
CL
LCL
1 98 228 374 520 666 812 958 1120 1298 1476 1654 1832 2010 2188 2366 2544 2722 2900 3078
Group
Number of groups = 3248
Center = 18.41392
StdDev = 7.168537
LCL = −3.091696
UCL = 39.91953
Number beyond limits = 180
Number violating runs = 783

b

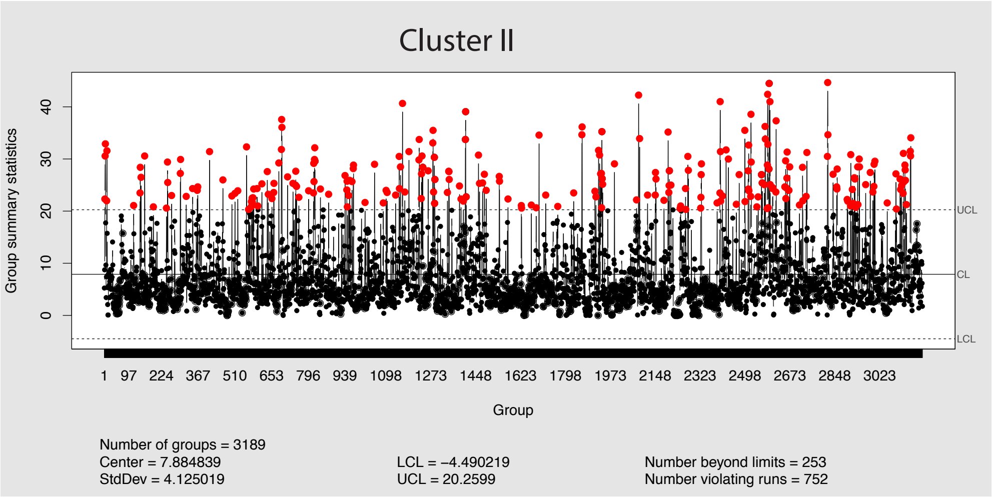
Cluster II
Group summary statistics
0
10
20
30
40
UCL
CL
LCL
1 97 224 367 510 653 796 939 1098 1273 1448 1623 1798 1973 2148 2323 2498 2673 2848 3023
Group
Number of groups = 3189
Center = 7.884839
StdDev = 4.125019
LCL = −4.490219
UCL = 20.2599
Number beyond limits = 253
Number violating runs = 752

Supplementary Figure S7 Analysis of mutational hotspots in members of cluster-I and cluster-II

Shewhart control charts showing the average SNVs frequencies for: (a) cluster-I strains and (b) cluster-II strains. Red and black dots indicate out of control (hotspots) and in-control SNV frequencies, respectively.

Supplementary Figure S8 Competition indices (CI) for 1218S *vs* 1218R during infection of whole animals (Japanese medaka)

The CI values were plotted as a log-trend for colonization of whole animals resulting in an average CI value = 0.27±0.28 as indicated.

The experiments were performed as previously described[51,67] (see also figure legend Fig S8).

Fig S8

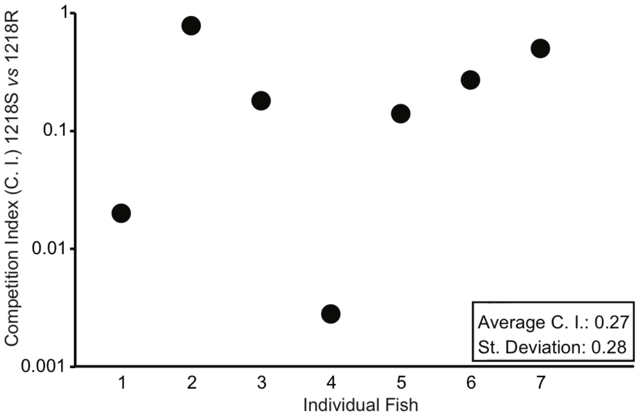

Figure legend S8

The Japanese medaka (*Oryzias latipes*)-*Mma* infection-model has previously been described[51,67]. Yellow Fever mosquito (*Aedes aegyti*) were employed as a vector to orally deliver *Mma* to a fish host[20]. Cultures for each of the two *Mma* strains (1218R and 1218S) were separately propagated and concentrations were validated by colony counts on 7H10 plates with 10% ADS and 0.5% glycerol, supplemented with cyclohexamide, polymixin B sulfate, and ampicillin[51]. Each fish was fed with five *Mma* infected larvae (~10^4 CFU) per "meal". Four larval meals were delivered on every other day for each fish and then fish were sacrificed after 90 days post infection. Fish were euthanized with a 0.1% solution of MS-222[51]. To reduce bacterial growth originating from the "fish surface" every sacrificed fish were incubated on ice in PBS (pH 7.4) supplemented with 100 µg/mL cycloheximide, 100 µg/mL ampicillin, 20 µg/mL polymixin B sulfate and 100 µg/mL gentamycin for 10 hrs (Mutoji and Ennis, unpublished method). Whole animals were washed and re-suspended (repeated four times) in 1 mL cold PBS. To determine whole-body infectious burdens each animal was homogenized and dilutions of homogenized tissues were spread on 7H10 plates supplemented with ADS, glycerol, cyclohexamide, polymixin B sulfate, and ampicillin (concentrations as above) and then incubated at 30°C for approximately 10 to 14 days. After incubation, plates were exposed to natural light for two days for the development of yellow pigmentation of *Mma* colonies and then monitored for the appearance of rough and smooth colony phenotypes. Rough and smooth colony phenotypes were quantified and photographed on plates, using a Nikon SMZ800 Stereoscopic Microscope[64,67]. The competition index (CI) of each fish was calculated as described elsewhere[67,68] with the following equation: [(CFU mutant output)/(CFU wild-type output)]/[(CFU mutant input)/(CFU wild-type input)]. In this calculation, 1218S was defined as the "mutant" and 1218R was referred to the as the "wild

type strain". When two strains are equivalent in virulence, the CI value is ≈1. In contrast, when mutant strain is less virulent with reduced whole-body colonization, the CI value is <1.

Ethics Statement

The fish handling procedures were conducted as prescribed by the National Institutes of Health-Biosafety in Microbiological and Biomedical Laboratories (NIH-BMBL) guidelines and were approved by both the Institutional Biosafety Committee and the Institutional Animal Care and Use Committee at the University of Louisiana (Animal Assurance Identification number A3029-01 and IACUC approval No. 2016-8717-029) were we abide by i) "The Guide for the care and use of laboratory animals, 8th edition, 2010", ii) "The US PHS policy on humane care and use of laboratory animals" and iii) "The US Government principles for the utilization and care of vertebrate animals used in testing, research and training".

67. Mutoji, K. N. & Ennis, D. G. Expression of common fluorescent reporters may modulate virulence for Mycobacterium marinum: Dramatic attenuatiom results from Gfp over-expression. *Comp Biochem Physiol C* **155,** 39–48 (2012).

68. Ruley, K. M., Ansede, J. H., Prichett, C. I., Talaat, A. M., Reimschuessel, R. & Trucksis, M. Identification of *Mycobacterium marinum* virulence genes using signature-tagged mutagenesis and the goldfish model of mycobacterial pathogenesis. *FEMS Microbiol Lett* **232**, 75-81 (2004).
